# Screening of the Pandemic Response Box library identified promising compound candidate drug combinations against extensively drug-resistant *Acinetobacter baumannii*

**DOI:** 10.1038/s41598-024-72603-9

**Published:** 2024-09-17

**Authors:** Nonlawat Boonyalai, Dutsadee Peerapongpaisarn, Chatchadaporn Thamnurak, Wilawan Oransathid, Nantanat Wongpatcharamongkol, Wirote Oransathid, Woradee Lurchachaiwong, John S. Griesenbeck, Norman C. Waters, Samandra T. Demons, Nattaya Ruamsap, Brian A. Vesely

**Affiliations:** 1 Department of Bacterial and Parasitic Diseases, Armed Forces Research Institute of Medical Sciences (AFRIMS), Bangkok, Thailand; 2 Biological Chemistry and Drug Discovery, Wellcome Centre for Anti-Infectives Research, University of Dundee, Dundee, UK; 3 Division of Global Health Protection, Thailand MoPH-US CDC Collaboration, Nonthaburi, Thailand

**Keywords:** Drug screening, Drug discovery, Microbiology

## Abstract

Infections caused by antimicrobial-resistant *Acinetobacter baumannii* pose a significant threat to human health, particularly in the context of hospital-acquired infections. As existing antibiotics lose efficacy against *Acinetobacter* isolates, there is an urgent need for the development of novel antimicrobial agents. In this study, we assessed 400 structurally diverse compounds from the Medicines for Malaria Pandemic Response Box for their activity against two clinical isolates of *A. baumannii*: *A. baumannii* 5075, known for its extensive drug resistance, and *A. baumannii* QS17-1084, obtained from an infected wound in a Thai patient. Among the compounds tested, seven from the Pathogen box exhibited inhibitory effects on the in vitro growth of *A. baumannii* isolates, with IC_50_s ≤ 48 µM for *A. baumannii* QS17-1084 and IC_50_s ≤ 17 µM for *A. baumannii* 5075. Notably, two of these compounds, MUT056399 and MMV1580854, shared chemical scaffolds resembling triclosan. Further investigations involving drug combinations identified five synergistic drug combinations, suggesting potential avenues for therapeutic development. The combination of MUT056399 and brilacidin against *A. baumannii* QS17-1084 and that of MUT056399 and eravacycline against *A. baumannii* 5075 showed bactericidal activity. These combinations significantly inhibited biofilm formation produced by both *A. baumannii* strains. Our findings highlight the drug combinations as promising candidates for further evaluation in murine wound infection models against multidrug-resistant *A. baumannii*. These compounds hold potential for addressing the critical need for effective antibiotics in the face of rising antimicrobial resistance.

## Introduction

*Acinetobacter baumannii*, classified as an aerobic Gram-negative pathogen, is widely recognized as a prominent cause of hospital-acquired infections and significant wound-related ailments^1^. These infections manifest diversely, encompassing conditions such as urinary tract infections, meningitis, bacteremia, gastrointestinal issues, as well as skin and soft tissue infections, including hospital-acquired and ventilator-associated pneumonia (HAP, VAP)^1,2^. Over the past decade, there has been a rise in treatment failures among patients infected with *A. baumannii*, coinciding with the global spread of *A. baumannii* strains resistant to multiple drugs, affecting regions worldwide, including Europe, North America, South America, Asia, and Southeast Asia^2,3^.

Multidrug-resistant (MDR) and extensively drug-resistant (XDR) *A. baumannii* present a formidable challenge in clinical settings. Carbapenems have conventionally served as the first-line drugs against these strains; however, the widespread use of carbapenems has led to an alarming increase in carbapenem-resistant *A. baumannii* (CRAB). Consequently, polymyxin (or colistin) has emerged as a last-resort treatment option for MDR and XDR *A. baumannii* infections, despite associated systemic toxicities such as nephrotoxicity and neurotoxicity^2,4^. Unfortunately, reports of increasing colistin resistance worldwide pose a further threat to effective treatment^2,5,6^.

Recognizing the critical nature of the situation, the World Health Organization (WHO) has designated CRAB as a high-priority antibiotic-resistant pathogen for the research and development of new antimicrobial agents^7^. Moreover, the Centers for Disease Control and Prevention (CDC) classifies CRAB as an "urgent threat," emphasizing the need for immediate attention and action in the US National Action Plan for Combating Antibiotic Resistant Bacteria and Department of Defense (DoD) guidelines, focusing on One-health and community-acquired infections (CAIs).

In Thailand, the prevalence of CRAB has surged to 70–80% in 2020, prompting a pressing need for the discovery of new drug candidates to address the growing crisis and control the spread of resistant strains^8^. Recognizing the complexity and length of the drug development process, our study aligns with the overarching goal of evaluating the Medicines for Malaria Venture's (MMV) Pandemic Response Box—a compilation of 400 drug-like compounds—as a potential source for novel antimicrobial agents^9^. Notably, previous investigations into the Pandemic Response Box have identified promising drug candidates for various infectious diseases, underscoring its potential significance in combating emerging threats^10–13^.

In this context, our primary objective is to evaluate the Pandemic Response Box collection against two extensively drug-resistant *A. baumannii* isolates. A previous screen of the Pandemic Response Box on other *A. baumannii* isolates identified five hits: MMV396785, MMV1578568, MMV1578574, MMV1578564, and MMV1479850^14^. Our screening confirms these findings, except for MMV1479850. Additionally, we identified three new hits: MMV1580854, MMV1578566, and MMV1634402. Furthermore, we investigate drug combinations of the identified compounds to discover potential synergistic effects, aiming to develop innovative and effective treatment strategies.

## Materials and methods

### Compounds

In the pursuit of antibacterial activity against extensively drug-resistant *A. baumannii*, the MMV Pandemic Response Box compound library (Medicines for Malaria Venture, MMV) was systematically screened. Comprising 400 structurally diverse compounds, the Pandemic Box includes 201 antibacterial, 153 antiviral, and 46 antifungal compounds^15^. These compounds, provided in 96-well plates at a concentration of 10 mM in dimethyl sulfoxide (DMSO), have either been marketed or are in various phases of drug discovery or development.

### Bacterial strains, culture conditions, and whole-genome sequencing

Extensively drug-resistant *A. baumannii* strains, namely QS17-1084 and 5075, were employed in this study. QS17-1084 was isolated from a wound infection of a patient at Queen Sirikit Naval Hospital, while *A. baumannii* 5075 was obtained from the Walter Reed Army Institute of Research, USA^16,17^. All *A. baumannii* strains were cultured in cation-adjusted Mueller Hinton broth (CAMHB) (Becton, Dickinson, USA). Whole-genome sequencing was performed using MiSeq or NextSeq benchtop sequencers (Illumina, Inc., Diego,CA) at the Multidrug-resistant Organism Repository and Surveillance Network (MRSN)^18^. The genome data of *A. baumannii* strains 5075 were deposited in GenBank under the accession number AHAH00000000^16^. The genomic sequence of QS17-1084 is available in NCBI database (BioProject No. PRJNA814829, BioSample No. SAMN42720897 and SRA No. SRR29910022)^17^.

### Pandemic Response Box compounds screening against *A. baumannii*

The initial 10 mM stock plates of the 400 compounds were diluted in 100% DMSO to a concentration of 1 mM. The bacterial growth inhibition assay followed Clinical and Laboratory Standards Institute (CLSI) microdilution method guidelines^19,20^. Bacterial colonies were incubated with 10 µM of each compound in cation-adjusted MH broth, and optical density at 600 nm (OD600) was measured. Percent growth of compound-treated *A. baumannii* was calculated using the following formula:

$$\frac{OD600\; of \;compound \; treated \;A. \;baumannii-OD600 \; of \; the \;blank \;well) \times 100}{(OD600 \;of \;DMSO\; treated \;A. \;baumannii-OD600 \;of\; the \;blank\; well)}$$

Compounds inhibiting bacterial growth by more than 50% were selected for further testing [Results section specifies the compounds].

### Structural similarity measurements

Structural similarity between Pandemic Response Box compound hits and commonly used antibacterial drugs was calculated using atom pair fingerprints (APfp)^21^. APfp values were determined and subjected to hierarchical clustering analysis using ChemMineR software^22,23^.

### Antimicrobial susceptibility testing of the selected compounds

Antimicrobial susceptibility testing (AST) of *A. baumannii* QS17-1084 and 5075 against known antibiotic panels was performed using the Phoenix platform (panel NMIC/ID504; BD Diagnostics, NJ, USA). We determined the minimal inhibitory concentrations (MICs) and minimal bactericidal concentrations (MBCs) of 7 selected MMV Pandemic response box compounds, doxycycline hydrochloride (WRAIR), and imipenem (US Pharmacopeia, Frederick, USA) were determined in triplicate using broth microdilution (BMD) using Clinical and Laboratory Standards Institute (CLSI) guidelines^21^. Additionally, methods were performed in accordance with the relevant guidelines and regulations of the Armed Forces Medical Research Institute of Medical Sciences Scientific Review Committee and Institutional Review Board.

For MICs determination, the compounds were diluted as twofold serial dilutions^20^. Bacteria were suspended in a clear glass tube containing with CAMHB before adjusting the inoculum suspension to 0.5 MacFarland standard using densitometer (Den-1, Biosan, USA). Subsequently, the inoculum suspension was prepared to a density yielding of the final bacteria concentration approximately 5 × 10^5^ CFU/ml. Twenty microliters of the inoculum were subjected to 96 well microtiter plates (Thermo Scientific, MA, USA) with 20 μL of a series of drug-like compounds and control antimicrobial agents, to make up the final volume of 40 μL (doxycycline and imipenem) concentrations, except the last well that was left for media only. Doxycycline and imipenem were also used as quality control drugs for *E. coli* ATCC 25,922 and *P. aeruginosa* ATCC 27853, respectively. *A. baumannii* incubation conditions were 35 ± 2 °C for 20–24 h while 16–20 h for *E. coli* and *P. aeruginosa*. All experimental sets were performed in triplicate.

For MBCs determination, the solution of the last clear MIC well of each selected compound was further incubated onto sheep blood agar (Becton, Dickinson, USA) and MacConkey agar (Becton, Dickinson, USA) before incubating at 37 °C in ambient air for 20–24 h. MBCs was determined by observing the bacterial growth on the sheep blood agar and MacConkey agar plates.

For IC_50_ (50% inhibitory concentration) determination, *A. baumannii* strains were treated with various concentrations of test compound and cultured according to the microdilution method above. IC_50_s were estimated by nonlinear regression analysis using GraphPad Prism (San Diego, CA, USA).

### Drug combinations

Fixed ratio combinations of various antimalarial drugs were performed as previously described with some modifications^24–26^. Stock solutions of the selected Pandemic response box compounds as well as control drugs doxycycline (DOX) (WRAIR), erythromycin (ERY) (Sigma-Aldrich, Missouri), ciprofloxacin (CIP) (Sigma-Aldrich, Missouri), and 7 compounds from the MMV Pandemic Response Box, according to their %inhibition, were prepared as follows: 2 mM and 4 mM DOX in 70% ethanol for *A. baumannii* 5075 and QS17-1084, respectively; 10 mM ERY in 70% ethanol, 10 mM CIP in 0.1 N HCl, and 5 mM MMV compound in DMSO. The total of 64 drug combinations were performed. The solutions for each drug were combined in ratios of 1 + 1, 1 + 3, 3 + 1, 1 + 4, 4 + 1, and 1 + 2; with each drug also tested alone. 20 µL of single and combination drug solutions were then introduced into 96-well plates to give a row with two-fold serial dilutions. 20 µL of *A. baumannii* with a final 5 × 10^4^ CFU per well were added, and the test plates were incubated for 20–24 h at 37 °C. Bacteria growth was measured by OD_600_ as described above and the individual 50% fractional inhibitory concentrations (FIC_50_) were determined as previously described^27^. Isobolograms were constructed by plotting the FIC_50_ of drug A against the FIC_50_ of drug B for each of the six drug ratios. A concave curve indicated synergy, a straight-line represented additivity and a convex curve indicated antagonism. To obtain numeric values for the interaction, results were expressed as the sum of the FIC_50A_ and FIC_50B_. The sum FIC_50_ (ΣFIC_50_) values indicate the kinds of interaction as follows: synergism when ΣFIC_50_ < 0.5; toward synergism when ΣFIC_50_ < 1; additive when ΣFIC_50_ = 1; toward antagonism when ΣFIC_50_ > 1; antagonism when ΣFIC_50_ > 2 to 4. The IC_50_s of each drug in the test combination were standardized by allocating the value of 1 to each drug that was tested alone and prorated values for each fixed concentration ratio.

### Time-kill assay

In vitro time-kill assay was performed to evaluate antibiotic efficacy of drug combinations compared to individual compounds. According to drug the combination assay, MUT056399, brilacidin, and their combinations were tested against *A. baumannii* QS17-1084, while MUT056399, eravacycline, and their combinations were tested against *A. baumannii* 5075. Each drug was prepared separately to the desired concentration based on MIC determined against each *A. baumannii* strain. In brief, equal volumes (100 µL) of drug alone or drug mixture and bacterial suspension were added to a sterile 96-well flat bottom plate (Corning, NY, USA). CAMHB medium was used to dilute drug(s) and bacteria. To prepare bacterial inoculum, bacterial culture was adjusted to 0.5 McFarland turbidity and diluted to 1:100 then inoculated into the wells containing drugs in duplicates. Plates were incubated in a 35 ± 2 °C incubator. Bacterial growth was observed by CFU count and OD_600_ measurement for 6 time points at 0, 2, 4, 6, 8, and 24 h incubation time. CFU count was performed by spreading the 10-folds serially diluted suspension onto tryptic soy agar plates. Results were expressed in CFU/mL. The limit of detection was set at 5 CFU/mL. The reduction in CFU/mL at 24 h was used to interpret results following the criteria; the reduction of ≥ 2 log_10_, but < 3 log_10_ was defined as bacteriostatic activities, and the reduction of ≥ 3 log_10_ was defined as bactericidal activities, relative to initial inoculum. For drug combination, the reduction of ≥ 2 log_10_ relative to the most active component alone was defined as synergistic activities^28^. Experiments were performed in two independent runs.

### Inhibition of biofilm formation

The effect of synergistic drug combinations were tested on the inhibition of biofilm formation of *A. baumannii* QS17-1084 and *A. baumannii* 5075. The concentrations of drugs were monitored to observe synergistic effects using a time-kill assay. Briefly, bacterial culture was adjusted to 0.5 McFarland turbidity in tryptic soy broth (TSB). Drugs were diluted in TSB with 1% glucose. Equal volumes (100 µL) of the 0.5 McFarland bacterial suspension and drug combination were added to a 96-well polystyrene non-treated, flat-bottom plate. This obtained a final concentration of 0.5% glucose in TSB. The respective strain of *A. baumannii* (without drugs) was used as a growth control and medium alone was used as a blank. In each run, *A. baumannii* ATCC19606 and *E. coli* ATCC25922 were included as positive and negative biofilm producing strains, respectively. Plates were incubated at 37 °C for 24 h with static condition. After incubation, the plates were gently washed three times with 300 µL of phosphate-buffered saline to remove planktonic and loosely adhered bacteria. The plates were fixed with 200 µL of methanol for 15 min, air dryed for 30 min, and stained with 175 µL of 0.5% crystal violet for 10 min. The plates were washed three times with 300 µL of distilled water and the crystal violet stain was solubilized by adding 175 µL of 33% acetic acid. After shaking for 10 min at room temperature, the solution was transferred to another plate and measured at OD_595_. Experiments were performed in three independent runs.

For result interpretation, the optical density cut-off (ODc) was defined as 3 standard deviation (SD) above mean OD of negative control. The degrees of biofilm formation were classified as follows: strong biofilm formation (OD > 4 × ODc), moderate biofilm formation (2 X ODc < OD ≤ 4 × ODc), weak biofilm formation (ODc < OD ≤ 2 × ODc), and non-biofilm formation (OD ≤ ODc). To calculate inhibition of biofilm formation, the absorbance values for the blank wells were subtracted from the values for the test wells to minimize background interference. Percentage of inhibition of biofilm formation was calculated as follows.

$$\text{Inhibition \;of \;biofilm \;formation }(\text{\%}) =1- \left[\frac{OD595 \;of \;compound\; treated\; A.\; baumannii}{OD595 \;of \;untreated \;A.\; baumannii} \right] \times 100$$

### Statistical analysis

Statistical analysis was performed using GraphPad Prism 10.0 (GraphPad Software, Inc., San Diego, CA, USA). The difference of data between groups was assessed by multiple t test, unless otherwise stated. Statistical significance was defined as a P value < 0.05.

## Results

### Phenotypic identification

**Fig. 1:**
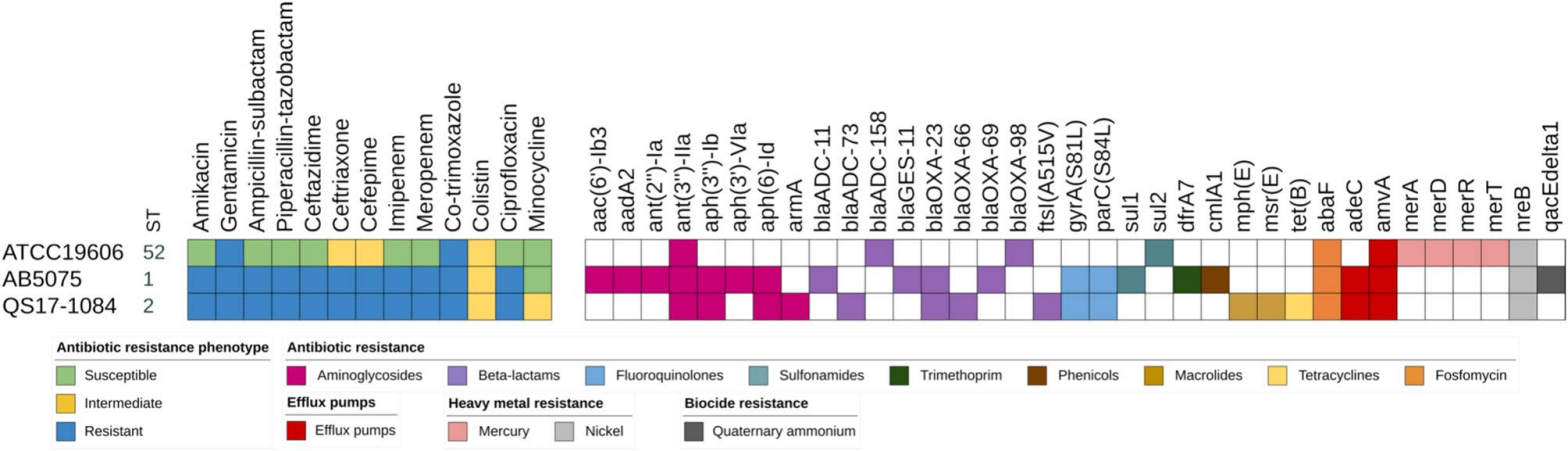
Characteristics of *A. baumannii* ATCC19606 (ATCC19606), *A. baumannii* 5075 (AB5075), and *A. baumannii* QS17-1084 (QS17-1084). Strain sequence type (ST) is indicated next to each strain. The assigned antimicrobial agent resistance phenotype is provided where the green, yellow and blue squares indicate a result of susceptible, intermediate, and resistant to the tested antimicrobial agents, respectively. The known antimicrobial agent resistant genes are provided in which each color represents each class or subclass of antimicrobial agent as well as mode of their inhibition. The figure was produced using the iTOL tool^31^.

Initially, we selected *A. baumannii* QS17-1084 since we are interested in using this strain of bacteria further in our animal wound model as part the WRAIR/AFRIMS drug development pipeline. *A. baumannii* 5075 has been previously employed in animal models^29,30^. *A. baumannii* QS17-1084, collected from the infected wound of a patient, was phenotypically identified as well as their antimicrobial susceptibility, in comparison to *A. baumannii* 5075, using the Phoenix M50 automated microbiology platform with NMIC/ID504 panel. Antimicrobial susceptibility testing (AST) results revealed *A. baumannii* QS17-1084 was resistance to almost all antimicrobial classes including aminoglycosides (amikacin and gentamicin), carbapenems (imipenem and meropenem), cephalosporins (ceftazidime, and cefepime), ciprofloxacin, piperacillin-tazobactam, and trimetroprim-sulfamethoxazole (Fig. 1), similar to *A. baumannii* 5075, except for tetracyclines (minocycline) in which *A. baumannii* QS17-1084 was intermediate while *A. baumannii* 5075 susceptible. In addition, both *A. baumannii* strains presented the MIC of 2 µg/ml towards colistin, indicative of colistin intermediate.

### Antimicrobial resistance genes detection

*A. baumannii* QS17-1084 was the further characterized through advance genome sequencing in comparison with *A. baumannii* ATCC19606 and 5075 (Fig. 1). *A. baumannii* QS17-1084 was classified in sequence type (ST) 2, while *A. baumannii* 5075 was ST1. Genes causing resistances to 9 antibiotic classes were identified. *A.baumannii* QS17-1084 carried genes causing resistance to aminoglycosides (*ant*(3″)*-IIa*, *aph(3″)-Ib*, and *aph(6)-Id* and *armA*), β-lactams (*bla*_ADC-73_, *bla*_OXA-23_, *bla*_OXA-66_, and *ftsI*(A515V)), fluoroquinolones (*gyrA*(S81L) and *parC*(S84L)), macrolides (*mphE* and *msrE*), tetracyclines (*tetB*), and fosfomycin (*abaF*). In addition, *adeC* and *amvA* involving in efflux pump over activity and causing reducing susceptibility or resistance to several antimicrobial classes such as β-lactam aminoglycosides, fluoroquinolones, tetracyclines, and erythromycin, respectively, as well as *nreB* gene generating resistance to heavy metals were also detected in *A. baumannii* QS17-1084. Similar to *A. baumannii* QS17-1084, *A. baumannii* 5075 harbored genes encoding resistance to aminoglycosides (*aac(6′)-Ib3*, *aadA2*, *ant(2″)-Ia*, *ant*(3″)*-IIa*, *aph(3″)-Ib*, *aph(3′)-*VIa, and *aph(6)-Id*), β-lactams (*bla*_ADC-11_, *bla*_GES-11_, *bla*_OXA-23_, and *bla*_OXA-69_), fluoroquinolones (*gyrA*(S81L) and *parC* (S84L)), sulfonamides (*sul1*), trimethoprim (*dfrA7*), phenicols (*cmlA1*), fosfomycin (*abaF*), heavy metal* (nreB*), and biocide (*qacEΔ1*). It is noted that *A. baumannii* 5075 was susceptible to tetracyclines.

### Identification of screening drugs against extensively drug-resistant *A. baumannii*

#### Primary screening

**Fig. 2:**
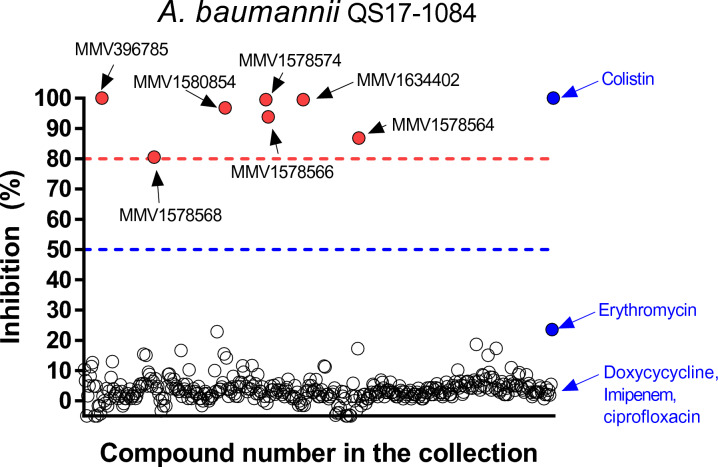
Antibacterial activities against *A. baumannii* QS17-1084 in the Pandemic Response Box collection at 10 µM concentration. Activities of known antibacterials are represented by blue circles [colistin (12.6 µM), erythromycin (12.8 µM), doxycycline (8.3 µM), imipenem (12.6 µM), and ciprofloxacin (9.4 µM)]. Most of the compounds had activities below the 50% growth inhibition cutoff. Seven of the most active compounds were selected for further tests based on their ability to inhibit the growth of *A. bamannii* QS17-1084 in the 80–100% range. These compounds are indicated by red circles.

Initially, a primary screen concentration of 10 µM of the Pandemic response box compounds was carried out against *A. baumannii* QS17-1084, using the CLSI microdilution method. As part of our target product profile compound with > 80% inhibitory effect are advanced for further investigation. The screening assay produced 7 compounds that had a > 80% inhibitory effect against both *A. baumannii* QS17-1084 and 5075 (Fig. 2 and Table 1). Five of the seven compounds with inhibitory activities including alexidine, gepotidacin, eravacycline, epetraborole, and brilacidin are reference compounds with known antifungal and antibacterial activity. Another two compounds, MUT056399 and MMV1580854, sharing a core structure of diphenyl ethers, inhibited 95% of the growth of *A. baumannii* 5075 while inhibited *A. baumannii* QS17-1084 to around 80–86% in the primary screen. All seven compounds inhibited the growth of *A. baumannii* QS17-1084 strain greater than erythromycin, doxycycline, imipenem, and ciprofloxacin (Fig. 2).

**Table 1:** Information on the compounds selected from the MMV Pandemic Response Box collection^a^.

MMV-ID (Trivial name)	Structure^b^	Original reported activity	Growth inhibition at 10 μM (%) (mean ± SD) (n = 3)	
			*A. baumannii*	
			QS17-1084	5075
MMV396785 (Alexidine)		Antifungal	100 ± 0.0	100 ± 0.0
MMV1578568 (Gepotidacin)		Antibacterial	80.5 ± 1.5	95.3 ± 3.5
MMV1580854		Antibacterial	96.8 ± 2.5	97.7 ± 1.9
MMV1578574 (Eravacycline)		Antibacterial	99.5 ± 0.4	100 ± 0.0
MMV1578566 (Epetraborole)		Antibacterial	93.8 ± 4.2	100 ± 0.0
MMV1634402 (Brilacidin)		Antibacterial	99.5 ± 0.4	100 ± 0.0
MMV1578564 (MUT056399)		Antibacterial	86.8 ± 2.9	95.4 ± 3.4

^a^MMV, Medicine for Malaria Venture.

^b^Structures were redrawn using ChemDraw Professional.

#### Hierarchical clustering analysis

Next we performed the hierarchical clustering analysis (HCA) of the 7 compounds and 4 commonly known antibacterial drugs, including ciprofloxacin, tetracycline, colistin and gentamicin (Fig. S1). The HCA revealed that gepotidacin, a first-in-class triazaacenaphthylene antibiotic inhibiting bacterial DNA replication, showed the maximum structural similarity with ciprofloxacin, a fluoroquinolone antimicrobial agent. Eravacycline, a synthetic halogenated tetracycline displayed close structural similarity with tetracycline, while MUT056399 was structurally closed to MMV1580854, suggesting that they may inhibit the same biological target, which is enoyl acyl carrier protein reductase (FabI). Epetraborole, a class of boron-heterocyclic antimicrobials targeting leucyl-tRNA synthetase (LeuRS)^32^ may relate to alexidine, a symmetrical alkyl bisbiguanide whereas brilacidin, an investigational new drug representing a new class of antimicrobial agents called host defense protein mimetics, exhibits the maximum structurally similarity with colistin (also known as polymyxin E) and disrupts bacterial cell membranes.

### Evaluation of antibacterial effects of the selected drugs on *A. baumannii*

To further understand the effect of screening drugs on *A. baumannii*, the MIC and MBC assays were utlized (Table 2). For *A. baumannii* QS17-1084, alexidine and brilacidin exhibited the lowest MIC values of 10 µM, while eravacycline and epetraborole provided the same MIC values of 20 µM. Gepotidacin and imipenem showed the MIC values of 40 and 50 µM, respectively. The compounds expressing the MIC value greater than 100 µM included doxycycline (133 µM), MMV1580854 and MUT056366 (> 160 µM). Unlike *A. baumannii* QS17-1084, the MIC values of the four compounds considerably decreased when tested against *A. baumannii* 5075. The MIC value of doxycycline was 133-fold decrease, followed by eravacycline (16 folds), epetraborole (fourfolds), and alexidine (twofolds). The highest MIC values were still from MMV1580854 and MUT056399 (> 160 µM). Most MBC values were similar to the MIC values except for epetraborole of *A. baumannii* QS17-1084 and imipenem of *A. baumannii* 5075 in which their MBC values were higher than their MIC values (> 160 µM for epetraborole and 100 µM for imipenem).

**Table 2:** Inhibitory and bactericidal properties of the selected MMV Pandemic response box compounds and control drugs.

Compound	Antibacterial activity (MICs and MBCs [μM]^a^			
	*A. baumannii* QS17-1084		*A. baumannii* 5075	
	MIC	MBC	MIC	MBC
Alexidine	10	10	5	5
Gepotidacin	40	40	40	40
MMV1580854	> 160	> 160	> 160	> 160
Eravacycline	20	20	1.25	1.25
Epetraborole	20	> 80	5	> 80
Brilacidin	10	10	10	10
MUT056399	> 160	> 160	> 160	> 160
Doxycycline	133	133	1	1
Imipenem	50	50	50	100

^a^MIC, minimum inhibitory concentration; MBC, minimum bactericidal concentration.

**Fig. 3:**
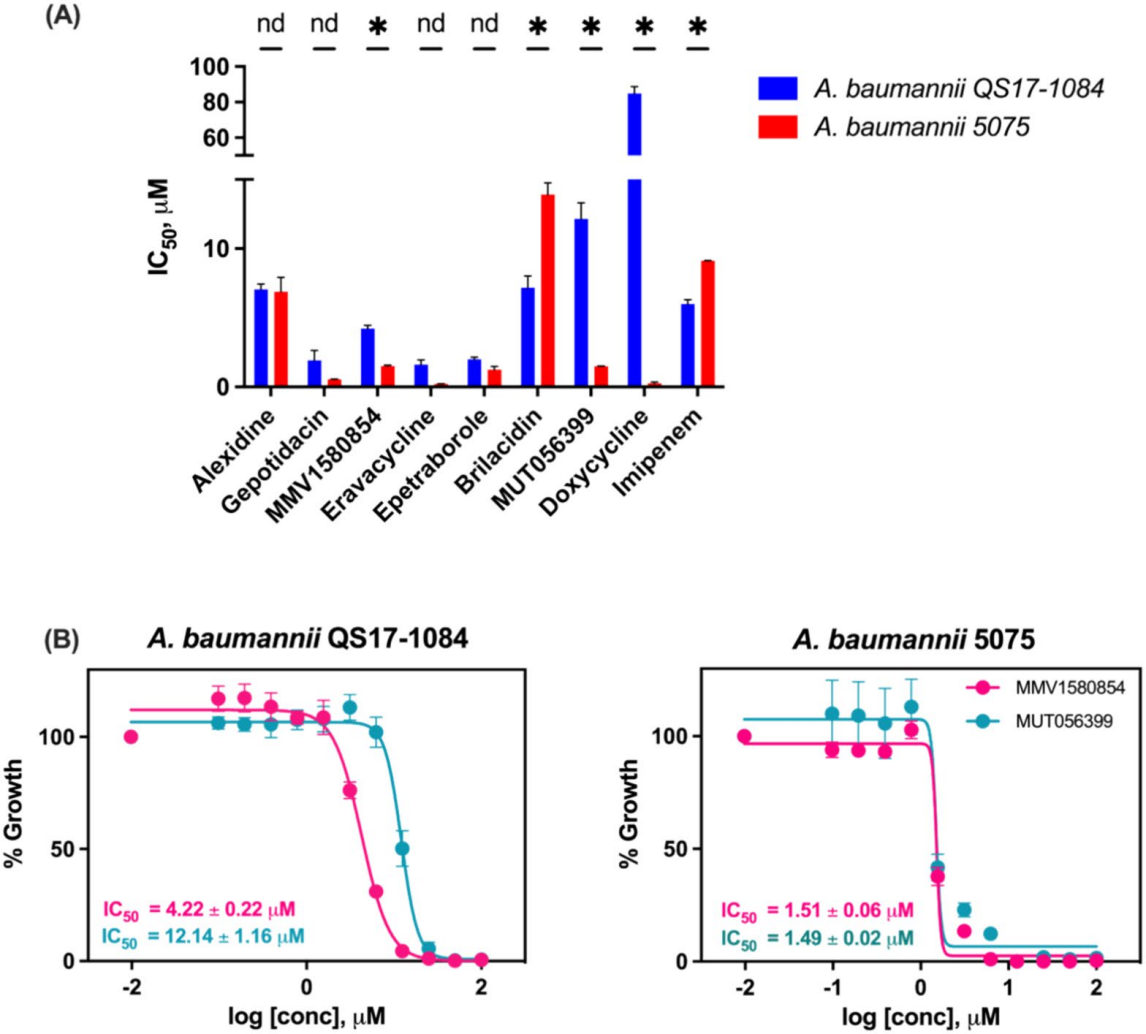
Inhibition of *A. baumannii *in vitro growth by 7 MMV Pandemic response box compounds. (**A**) Half maximal inhibitory concentration (IC_50_) of the compounds against *A. baumannii* QS17-1084 and 5075. (**B**) IC_50_ comparison of MMV1580854 and MUT056399 against *A. baumannii* QS17-1084 and 5075. Statistically significant differences calculated by multiple unpaired t test are indicated with one (P < 0.05) and nd for no difference.

To better understand drug sensitivity, we subsequently determined the IC_50_ values, the concentration of drug required for 50% inhibition, of 9 compounds against both *A. baumannii* QS17-1084 and 5075 (Fig. 3A). For *A. baumannii* QS17-1084, 3 compounds, including gepotidacin, eravacycline, and epetraborole, showed a relatively good drug sensitivity with the IC_50_ values ranging from 1.61 to 1.99 µM. Four compounds including alexidine, MMV1580854, brilacidin, and imipenem provided a comparable IC_50_ values ranging from 4.22 to 7.17 µM, while MUT056399 and doxycycline had the elevated IC_50_ values of 12.14 and 84.87 µM, respectively. When the compounds were tested against *A. baumannii* 5075, the IC_50_ values of 5 compounds, including MMV1580854, brilacidin, MUT056399, doxycycline, and imipenem, were altered significantly compared with *A. baumannii* QS17-1084 (Fig. 3A), while those of alexidine, gepotidacin, eravacycline, epetraborole were not significantly changed. Of five compounds, MMV1580854, MUT056399 and doxycycline showed an increase in drug sensitivity towards *A. baumannii* 5075. Doxycycline showed the 314-fold reduced IC_50_ value, followed by MUT056399 (8.20-fold decrease), and MMV1580854 (2.71-fold decrease). Two compounds, brilacidin and imipenem, exhibited the increased IC_50_ values against *A. baumannii* 5075 around 1.94- and 1.52-fold increases, respectively. In addition to IC_50_ values, IC_99_ values of each compound were also determined (Table S1). The IC_99_ concentration which is approximately 100-fold the IC_50_ concentration is used when complete inhibition is required. The IC_99_ values of some compounds against *A. baumannii* QS17-1084 were comparable to MIC values, including alexidine, brilacidin, while for the rest of the compounds, the IC_99_ values were smaller than the MIC values. In the case of *A. baumannii* 5075, the IC_99_ values of eravacycline, epetraborole, and doxycycline were close to the MIC values. While the IC_99_ values of MMV1580854, MUT056399, and imipenem were less than the MIC values, those of alexidine, gepotidacin and brilacidin were higher than the MIC values in *A. baumannii* 5075. Since MMV1580854 shares chemical structure similarity with MUT056399, we closely compared their IC_50_ values (Fig. 3 B). When both compounds were tested against *A. baumannii* 5075, the IC_50_ values of both compounds were not significantly different (1.51 µM for MMV1580854 and 1.49 µM for MUT056399. However, when tested against *A. baumannii* QS17-1084, the IC_50_ value of MMV1580854 was 2.87-fold decreased compared to the IC_50_ value of MUT056399, indicating that MMV1580854 was more potent than MUT056399 when treated against *A. baumannii* QS17-1084.

### Drug combination effects on *A. baumannii*

**Fig. 4:**
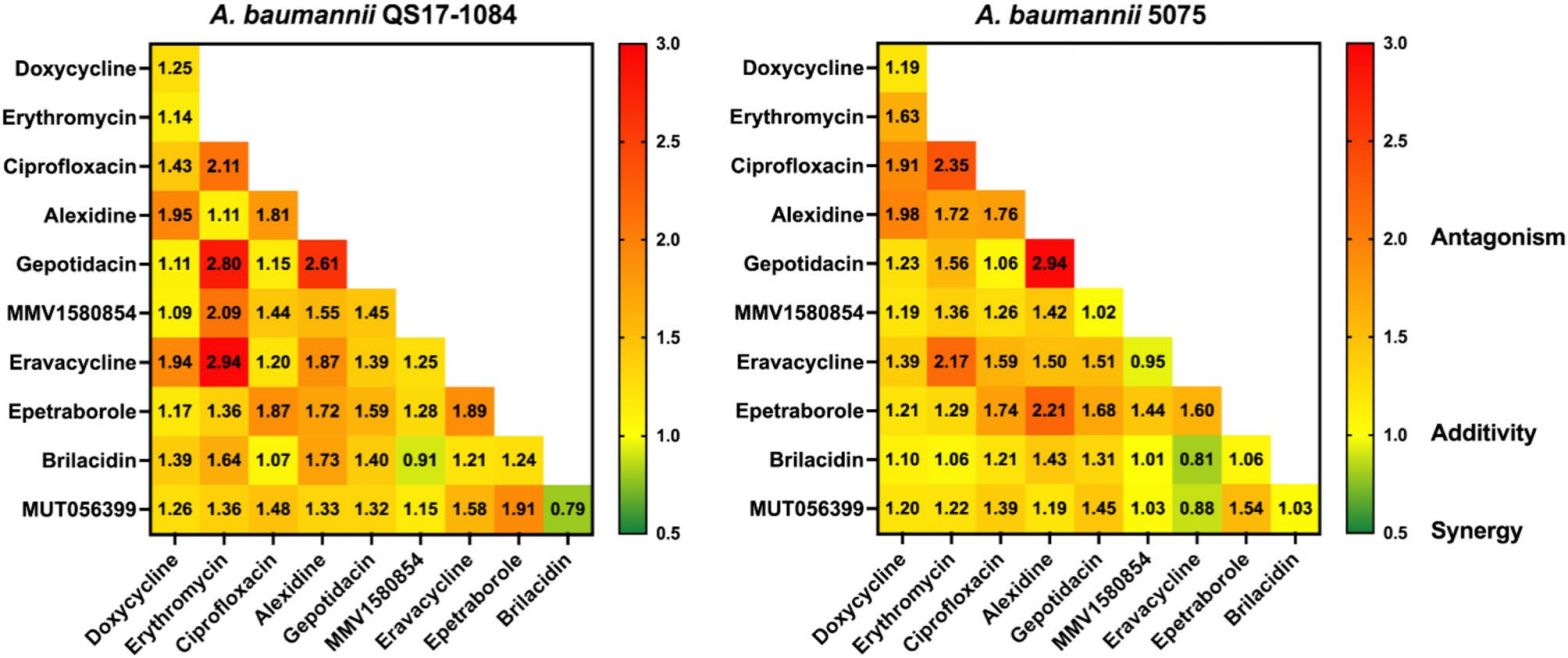
Drug interactions in *A. baumannii*. Forty-six drug combinations were carried out against both *A. baumannii* QS17-1084 and 5075. The numbers represent ΣFIC_50_ (50% Fractional Inhibitory Concentrations) values: ΣFIC_50_, synergism when ΣFIC_50_ ≤ 0.5; toward synergism when ΣFIC_50_ < ; additive when ΣFIC_50_ = 1; toward antagonism when ΣFIC_50_ > 1; antagonism when ΣFIC_50_ ≥ 2 to 4. The values show the mean ± S.D. of 3 independent assays for each *A. baumannii* strain.

Finding novel antimicrobial agents and therapies based on synergistic combinations are crucial to battle drug-resistant bacteria. Of 7 potent compounds from the Pandemic Response Box, and 3 known antibiotics, the 46 drug combinations were investigated (Fig. 4) to evaluate the synergistic effects using previously published methods^25^. Two drug combinations (brilacidin-MMV1580854, and brilacidin-MUT056399) showed toward synergistic effects (ΣFIC_50_ < 1) on *A. baumannii* QS17-1084, while 3 combinations (eravacycline-MMV1580854, eravacycline-brilacidin, and eravacycline-MUT056399) had toward synergistic activity against *A. baumannii* 5075. It is interesting that out of the 6 combinations with synergism, they were stemmed from 4 compounds including brilacidin, eravacycline, MMV1580854, and MUT056399, the latter two of which shared similar chemical scaffold. Antagonistic effects (ΣFIC_50_ > 1) were also observed in 5 drug combinations for *A. baumannii* QS17-1084 and 4 drug combinations for *A. baumannii* 5075. Three drug combinations showing antagonistic interactions in both *A. baumannii* strains were ciprofloxacin-erythromycin, gepotidacin-alexidine, and eravacycline-erythromycin.

### Time-kill assay for assessing the efficacy of drug combinations

**Fig. 5:**
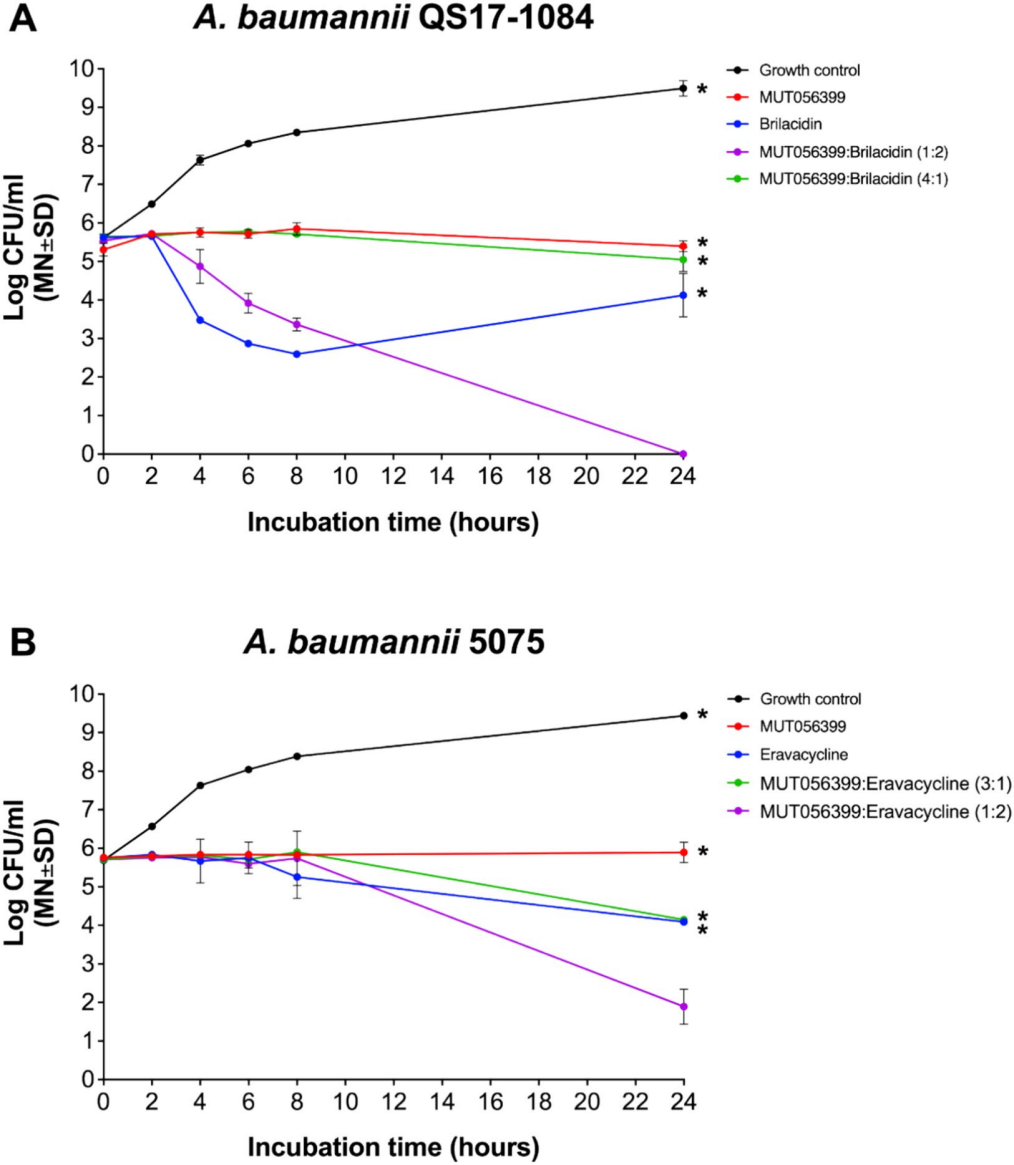
Time-kill curve. (**A**) *A. baumannii* QS17-1084 incubated without antibiotic (growth control), with MUT056399, brilacidin, MUT056399:brilacidin (ratio 1:2), and MUT056399:brilacidin (ratio 4:1). (**B**) *A. baumannii* 5075 incubated without antibiotic (growth control), with MUT056399, eravacycline: MUT056399:eravacycline (ratio 3:1), and MUT056399 : eravacycline (ratio 1:2). *P < 0.05 was analyzed by Dunnett's multiple comparisons test to compare between drug combination (MUT056399 and brilacidin at the ratio 1:2 for *A. baumannii* QS17-1084; and MUT056399 and eravacycline at ratio 1:2 for *A. baumannii* 5075) versus other tested compounds and growth control.

According to drug combination results, in vitro time-kill assay was performed to evaluate drug combination antibiotic efficacy. The drug combination with synergistic effect was chosen (Fig. 4). For *A. baumannii* QS17-1084, MUT056399 and brilacidin at the ratios of 1:2 (low FIC_50_) and 4:1 (high FIC_50_), respectively, were selected (Table S2), whereas MUT056399 and eravacycline were chosen at the ratios of 1:2 (high FIC_50_) and 3:1 (low FIC_50_) (Table S3) to test against *A. baumannii* 5075. Table S4 summarizes the concentration used in the assay. Figure 5 shows time-kill curves of *A. baumannii* strains against both individual drug and combined drugs. According to Fig. 5A, *A. baumannii* QS17-1084 treated with the combination of MUT056399 and brilacidin at the ratio 1:2 shows the complete inhibition after 24 h with the reduction of 5.5 log_10_ CFU/mL bacteria compared to the initial count (P value < 0.0001) and the reduction of 4 log_10_ CFU/mL compared to the most active compound alone, 10 µM brilacidin, (P value < 0.0001). The observed reduction indicated bactericidal and synergistic activities of MUT056399 combined with brilacidin at a ratio of 1:2. In contrast, the combination of MUT056399 and brilacidin at the ratio 4:1 did not profoundly inhibit the QS17-1084 strain and showed a reduction by 10.6% after 24 h (5.05 log_10_ CFU/ml) compared to initial inoculum (5.65 log_10_ CFU/ml) (Fig. 5A). In addition, CFU/mL of the drug combination MUT056399 with brilacidin at the ratio 4:1 was not significantly different compared to each drug alone. Furthermore, MUT056399 and brilacidin alone as well as their combination at the ratio 4:1, showed neither bacteriostatic nor bactericidal effects against *A. baumannii* QS17-1084.

For *A. baumannii* 5075, the combination of MUT056399 and eravacycline exhibited a significant reduction in CFU/mL at the ratio of 1:2 with the reduction of 3.8 log_10_ CFU/mL) from the initial inoculum after 24 h (P value < 0.0001; Fig. 5B) and with the reduction of 2.2 log10 CFU/mL) in comparison to eravacycline alone (P value < 0.0001; Fig. 5B), shown as the most active compound. In addition, this drug combination at the ratio 1:2 displayed the synergistic and bactericidal activities. However, it was observed that combination of MUT056399 and eravacycline at the ratio of 3:1 did not present bactericidal activities. It seems that this minimal inhibitory effect was caused by eravacycline alone (Fig. 5B).

### Inhibition of biofilm formation

**Fig. 6:**
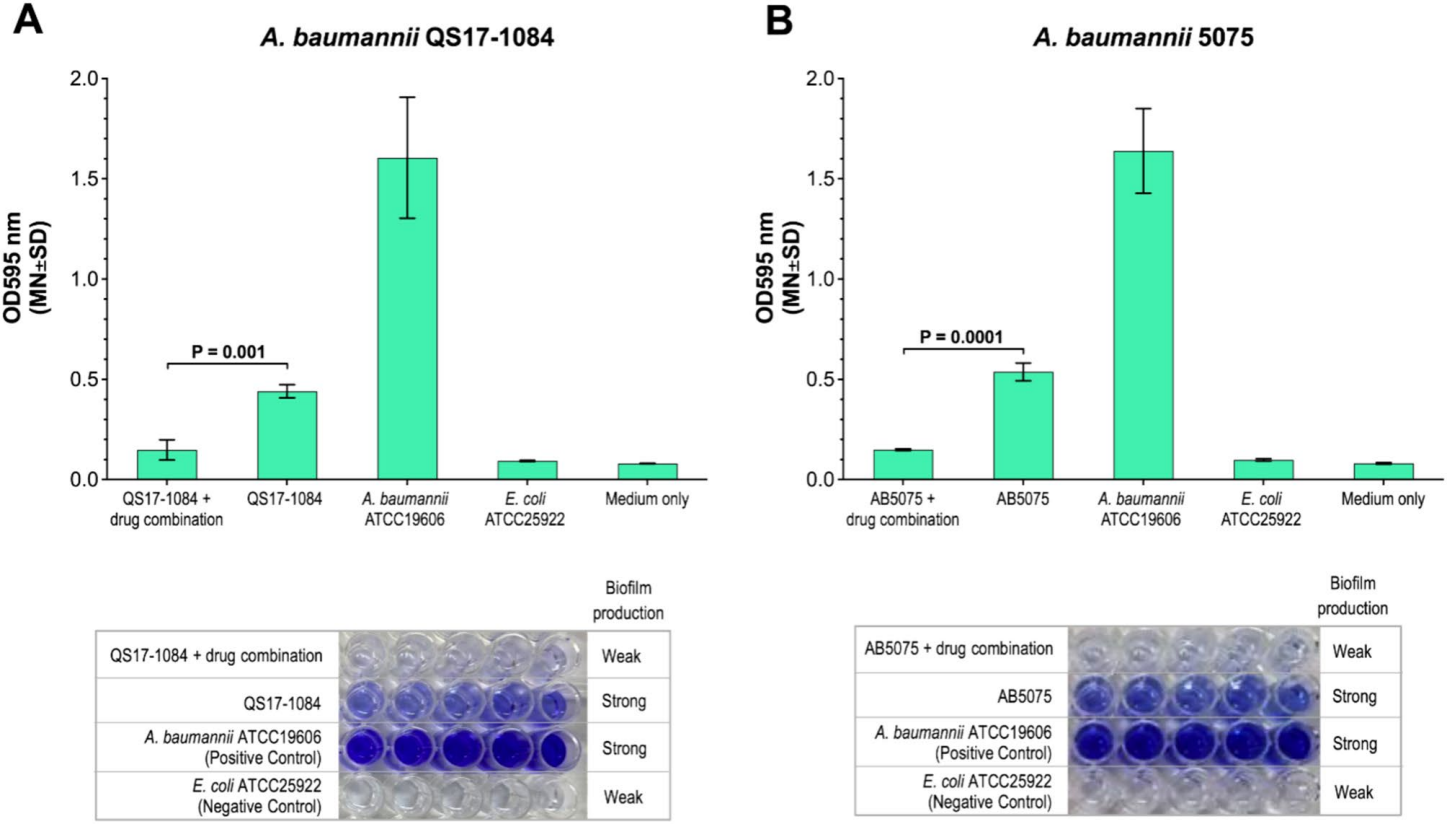
Effects of drug combination on preventing biofilm formation. (**A**) *A. baumannii* QS17-1084 was treated with MUT056399 and brilacidin at the ratio 1:2. (**B**) *A. baumannii* 5075 was incubated with MUT056399 and eravacycline at ratio 1:2. The drug concentrations were similar to those used in the time-kill assay. *A. baumannii* ATCC19606 and *E. coli* ATCC25922 were included as positive and negative biofilm producing strains, respectively. P < 0.05 was analyzed by using Student’s t-test. The representative images are biofilms stained by crystal violet.

The ability of drug combinations in inhibiting biofilm formation of *A. baumannii* QS17-1084 and *A. baumannii* 5075 was observed using crystal violet staining. Generally, both *A. baumannii* QS17-1084 and *A. baumannii* 5075 could produce biofilms but less than a positive control *A. baumannii* ATCC19606 (Fig. 6). It was revealed that the combination of MUT056399 and brilacidin (ratio 1:2) significantly inhibited biofilm formation of *A. baumannii* QS17-1084 and *A. baumannii* 5075, respectively in relative to bacteria alone without drug treatment (Fig. 6A and B). Percent inhibition of biofilm formation at 24 h was shown 80% and 85% when treated *A. baumannii* QS17-1084 and *A. baumannii* 5075, respectively with drug combination. Further, biofilm formation of both strains was profoundly inhibited after 24 h drug exposure, which the detected biofilms were presented in a similar manner to negative control strain, *E. coli* ATCC25922 (Fig. 6A and 6B).

## Discussion

The purpose of this work is to find inhibitors against extensively drug-resistant *A. baumannii*, which is a prominent member of ESKAPEE group of pathogens, and primarily associated with wound and burn infections and ventilator-associated pneumonia^33^. The 400 MMV Pandemic Response Box compounds were screened, and 7 compounds were selected and further evaluated through phenotypic experiments against 2 extensively drug-resistant *A. baumannii* strains, including QS17-1084 and 5075. In addition, we showed that the combinations of brilacidin and diphenyl ether derivatives, and eravacycline and brilacidin or diphenyl ether derivatives showed synergistic effects on *A. baumannii* QS1084 and 5075, respectively.

*A. baumannii* remains a heterogeneous species with over 1,380 different sequence types (STs) identified by traditional Pasteur scheme multilocus sequence typing (MLST)^34,35^. *A. baumannii* 5075 and QS17-1084 belonged to ST1 and ST2, respectively. By comparing with *A. baumannii* 5075, *A. baumannii* QS17-1084 had *ant(3″)-IIa*, *aph(3″)-Ib*, *aph(6)-Id* genes that encode aminoglycoside adenylyltransferase and aminoglycoside phosphotransferase, and *armA* gene that encodes 16S rRNA methyltransferase conferring high level resistance of various aminoglycosides^36^. The presence of these genes is well correlated with the drug susceptibility test in which both *A. baumannii* QS17-1084 and 5075 are resisted to amikacin and gentamicin (Fig. 1). *A. baumannii* QS17-1084 may resist to β-lactams via Class C and D β -lactamases as well as altering penicillin-binding proteins (PBP3) while *A. baumannii* 5075 may employ Class A, C and D β-lactamases for β-lactams resistance. The *bla*_ADC_ belongs to class C β-lactamases. The ADC β-lactamase are cephalosporinase with resistance extended-spectrum cephalosporins^37^. The *bla*_oxa_ genes, classified for class D β-lactamases resistance, were reported in most of carbapenemase producing *A. baumannii* in several studies in Southeast Asia, including Thailand^38^, Vietnam^39^, Malaysia^40^, China^41^ as well as in Europe^42^. The *bla*_oxa-66_ was grouped into intrinsic *bla*_oxa-51-like_ oxacillinase gene found in *A. baumannii*^37^. Both *A. baumannii* strains in this study showed resistance to ciprofloxacin because they harbored the mutations on *gyrA* and *parC* genes. The most distinction of these two *A. baumannii* strains is that *A. baumannii* QS17-1084 contained genes that confer resistance to macrolides and tetracyclines, so it showed intermediate against minocycline (Fig. 1) and elevated MIC and IC_50_ values (Table 2 and Fig. 3).

The screening of 400 diverse compounds identified seven compounds (Table 1 and Fig. 2), including alexidine, gepotidacin, MMV1580854, eravacycline, epetraborole, brilacidin, MUT056399. Alexidine is an anticancer drug, targeting a mitochondrial tyrosine phosphatase, PTPMT1, in mammalian cells and causing mitochondrial apoptosis^43^. It also inhibited planktonic growth, prevented biofilm formation as well as killing biofilms formed by diverse fungal organisms^44^. Alexidine dihydrochoride was also identified in a quantitative high throughput screen (qHTS) against *A. baumannii* 5075 with the IC_50_ of 29.02 µM. Gepotidacin is a novel triazaacenaphtylene antibiotic with inhibiting DNA gyrase and topoisomerase IV. Its activity was shown against most strains of *E. coli* and *Staphylococcus saprophyticus*^45^. MMV1580854 and MUT056399, which are diphenyl ether derivatives, have been shown as inhibitors against enoyl-acyl carrier protein reductase (FabI)^46,47^. The IC_50_ values of MMV1580854 and MUT056399 against*S. aureus* FabI was 56 and 12 nM, respectively and the MIC value of MMV1580854 against *S. aureus* CIP54,14 was about eightfold higher than that of MUT056399^47^. In our study, while the IC_50_ values of both MMV1580854 and MUT056399 against *A. baumannii* 5075 were very close (Table S1), MMV1580854 seemed to be more potent than MUT056399 against *A. baumannii* QS17-1084. Eravacycline, a newly developed, fully synthetic tetracycline derivative with broad-spectrum activity against extended spectrum β-lactamase producing *Enterobacteriaceae* and *Acinetobacter*^48^, inhibits bacterial protein synthesis through binding to the 30S ribosomal subunit. Eravacycline exhibited greater activity than the comparators of the tetracycline class with the MIC_50_ of 0.5 mg/L against multidrug-resistant, carbapenem non-susceptibility *A. baumannii* isolates.^49^. Since there is no information on clinical breakpoints against *A. baumannii*, Deolankar et al. suggested that the MIC values (< 4 μg/mL) of eravacyclines were effective against their 19 *A. baumannii* patient isolates^50^. In this study, the MIC value of eravacycline against *A. baumannii* 5075 was 1.25 μM (or 0.79 μg/mL), while that against *A. baumannii* QS17-1084 was 20 μM (or 12.6 μg/mL), which may be accounted for the presence of *tet*(B) gene. Cheng et al. screened 25 antibiotics commonly used for infections by gram negative bacteria and found that nine compounds could inhibit the growth of *A. baumannii* 5075 in concentration dependence (IC_50_ ranging from 0.39 to 66.5 µM)^51^. Tigecycline, a derivative of the tetracycline, was the most potent with the IC_50_ of 0.15 µM. This was in accordance with our result that eravacycline was the most potent with the IC_50_ of 0.22 µM^51^. Epetraborole, a new class of leucyl-tRNA synthetase inhibitors, showed potent activity against a broad range of Gram-negative bacteria^52,53^. Epetraborole demonstrated activity against wild-type *A. baumannii* (MIC_50_ of 2 μg/mL) while it was less active against multidrug-resistant *A. baumannii* (MIC_50_ of 8 μg/mL)^54^. In our study, epetraborole showed good activity against both isolates (MIC < 8 μg/mL) but more potent against *A. baumannii 5075* (MIC = 5 μM or 1.37 μg/mL) compared to a greater resistant *A. baumannii* QS17-1084 (MIC = 20 μM or 5.47 μg/mL). In addition, epetraborole showed potent activity against *Mycobacterium abscessus* and the combination of epetraborole and norvaline improved the in vivo efficacy compared to epetraborole alone^54,55^. Brilacidin, a small-molecule arylamide mimics of antimicrobial peptides (AMPs), has exhibited potent bactericidal activity against drug-resistant and -susceptible strains of multiple Gram-negative and Gram-positive pathogens^56,57^. Brilacidin caused membrane depolarization in *Staphylococcus aureus*^58^ and also used to be evaluated as an ocular anti-infective^59^. In addition, brilacidin showed the inhibitory activity against SARS-CoV-2^60–62^ and human pathogenic fungi^63^. Recently, MMV1580854, MUT056399, eravacycline, epetraborole, and MMV1579788 exhibited a higher percentage (> 90%) of *Klebsiella pneumoniae* growth inhibition^64^. This suggested the shared targets of the compounds between *A. baumannii* and *K. pneumoniae*. With respect to our IC_50_s of the compounds against both *A. baumannii* strains (Fig. 3), it was noticeable that some compounds were particularly potent against one strain. Doxycycline was less potent against *A. baumannii* QS17-1084. This may be due to the fact that *A. baumannii* QS17-1084 harbors tetracycline and macrolide resistant gene. Both strains harbor different beta-lactam resistant genes, and this might be accounted for the difference in the IC_50_ values against imipenem. In terms of MMV1580854, brilacidin, and MUT056399, there is no confirmed resistant genes against these compounds. However, our results showed that *A. baumannii* QS17-1084 showed higher IC_50_ values against both MMV1580854 and MUT056399, which share the same chemical scaffold. It might be worthwhile to check if there are mutations in *fab1* gene of both strains since it was hypothesized that these two compounds should share the same target which is FabI.

In terms of drug combination, the combinations of among MMV1580854, MUT056300, brilacidin and eravacycline provided toward synergistic effects against our tested *A. baumannii* isolates. The synergism when brilacidin combined with either MMV1580854 or MUT056399 could promote disrupting bacterial cell membranes and inhibiting fatty acid synthesis whereas when the combination of eravacycline combined with either MMV1580854 or MUT056399 could target for inhibiting both protein and fatty acid synthesis. The combination of eravacycline and brilacidin might disrupt protein synthesis and bacterial cell membranes. Brilacidin showed synergistic antiviral activity when combined with remdesivir^60^. Checkboard testing of one of the FabI inhibitors, sharing the similar structures as MMV1580854 and MUT056399 with known antibacterial agents against *A. baumannii* isolates revealed that the best effect was observed when the FabI inhibitor combined with colistin^65^ The combination effects of eravacycline with other standard-of-care antibiotics were carried out against XDR *A. baumannii* and the combination between eravacycline and amikacin showed additive and synergistic ^50^. The time-kill assay clearly confirmed the great efficacy of drug combinations in comparison to individual drugs. Bactericidal activities were observed significantly when drug combinations were used. The similar synergistic killing effect was also seen when econazole and colistin combination was treated to multidrug-resistant *A. baumannii*^66^.

## Conclusion

We have identified 7 drug candidates that significantly suppressed the growth of the extensively drug-resistant *Acinetobacter baumannii* 5075 and QS17-1084 strains. We also found three pairs and two pairs of two drug combinations that exhibited toward synergistic effect against *Acinetobacter baumannii* 5075 and QS17-1084, respectively. These combinations arose from brilacidin, eravacycline and two triclosan derivatives, including MMV1580854 and MUT056399. Regards to time-kill and biofilm assays, the combinations of MUT056399 and brilacidin or eravacycline will be selected for the efficacy test in the chronic wound infection mice model.

## Supplementary Information

Supplementary Figure S1.

Supplementary Table S1.

Supplementary Table S2.

Supplementary Table S3.

Supplementary Table S4.

## Data Availability

All data supporting the findings of this study are available within the paper and its Supplementary Information.
