# Supplementary material for: Screening of the Pandemic Response Box library identified promising compound candidate drug combinations against extensively drug-resistant Acinetobacter baumannii
Source: Sci Rep. 2024 Sep 17;14:21709. doi: 10.1038/s41598-024-72603-9 (PMC11408719; doi:10.1038/s41598-024-72603-9)
Supplement: Supplementary file 1 — Supplementary Figure S1. [file 41598_2024_72603_MOESM1_ESM.docx]

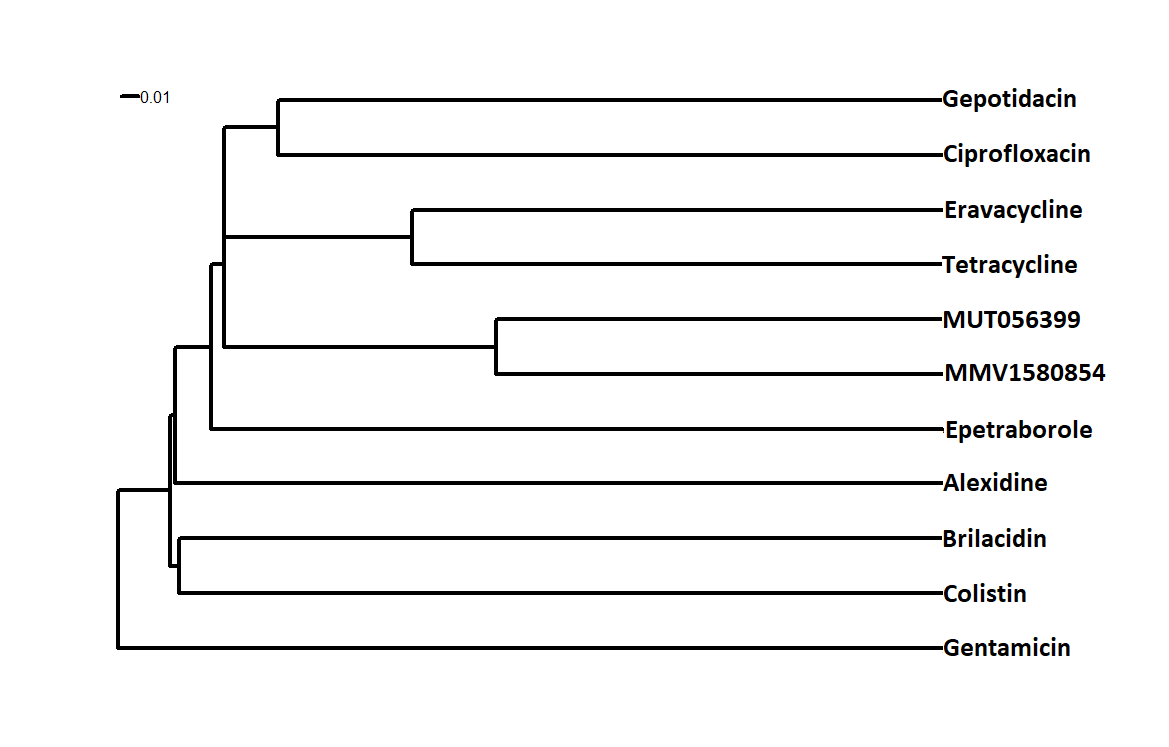


**Figure S1.** Hierarchical clustering analysis. The analysis was performed using ChemmineR software and highlights the structural similarities between the Pandemic response box compounds with anti- *A. baumannii* activity and commonly known antibacterial drugs (ciprofloxacin, tetracycline, colistin and gentamicin). Dissimilarity is reported using a scale unit of 0.01.
