# Supplementary material for: Screening of the Pandemic Response Box library identified promising compound candidate drug combinations against extensively drug-resistant Acinetobacter baumannii
Source: Sci Rep. 2024 Sep 17;14:21709. doi: 10.1038/s41598-024-72603-9 (PMC11408719; doi:10.1038/s41598-024-72603-9)
Supplement: Supplementary file 2 — Supplementary Table S1. [file 41598_2024_72603_MOESM2_ESM.docx]

**Table S1.** IC_50_ and IC_99_ value of test compounds

| **Compound** | ***A. baumannii* QS17-1084** | |  | ***A. baumannii* 5075** | |
| --- | --- | --- | --- | --- | --- |
|  | **IC_50_ (µM)** | **IC_99_ (µM)** |  | **IC_50_ (µM)** | **IC_99_ (µM)** |
| Alexidine | 7.05 ± 0.40 | 8.87 ± 0.24 |  | 6.89 ± 1.03 | 8.04 ± 0.66 |
| Gepotidacin | 1.92 ± 0.71 | 16.24 ± 4.71 |  | 0.56 ± 0.02 | 51.02 ± 15.48 |
| MMV1580854 | 4.22 ± 0.22 | 25.28 ± 8.13 |  | 1.51 ± 0.06 | 2.91 ± 1.30 |
| Eravacycline | 1.61 ± 0.34 | 3.48 ± 0.97 |  | 0.22 ± 0.03 | 0.36 ± 0.11 |
| Epetraborole | 1.99 ± 0.16 | 5.87 ± 0.92 |  | 1.24 ± 0.24 | 4.90 ± 0.38 |
| Brilacidin | 7.17 ± 0.85 | 8.45 ± 0.05 |  | 13.91 ± 1.08 | 21.37 ± 4.17 |
| MUT056399 | 12.14 ± 1.16 | 33.98 ± 3.46 |  | 1.49 ± 0.02 | 2.19 ± 0.39 |
| Doxycycline | 84.87 ± 3.79 | N/A |  | 0.27 ± 0.08 | 0.77 ± 0.21 |
| Imipenem | 5.99 ± 0.32 | 14.73 ± 1.06 |  | 9.11 ± 0.03 | 13.15 ± 0.29 |
