# Supplementary material for: Screening of the Pandemic Response Box library identified promising compound candidate drug combinations against extensively drug-resistant Acinetobacter baumannii
Source: Sci Rep. 2024 Sep 17;14:21709. doi: 10.1038/s41598-024-72603-9 (PMC11408719; doi:10.1038/s41598-024-72603-9)
Supplement: Supplementary file 3 — Supplementary Table S2. [file 41598_2024_72603_MOESM3_ESM.docx]

**Table S2.** the 50% fractional inhibitory concentrations (FIC_50_) of MUT056399 (Drug A) and brilacidin (Drug B) combination against *A. baumannii* QS17-1084

| **Drug A : Drug B** | **Sum of FIC's (1st)** | | **Sum of FIC's (2nd)** | | **Sum of FIC's (3rd)** | **Mean** | **SD** |
| --- | --- | --- | --- | --- | --- | --- | --- |
| **RATIOS** | **FIC_50_ Drug A + FIC_50_ Drug B** | | **FIC_50_ Drug A + FIC_50_ Drug B** | | **FIC_50_ Drug A + FIC_50_ Drug B** |  |  |
| 1 to 1 | 0.774 | | 0.666 | | 1.086 | 0.842 | 0.22 |
| 1 to 3 | 0.711 | | 0.594 | | 0.764 | 0.690 | 0.09 |
| 3 to 1 | 0.857 | | 0.752 | | 0.740 | 0.783 | 0.06 |
| 1 to 4 | 0.745 | | 0.592 | | 0.822 | 0.720 | 0.12 |
| 4 to 1 | 1.251 | | 1.005 | | 0.880 | 1.045 | 0.19 |
| 1 to 2 | 0.699 | | 0.559 | | 0.663 | 0.640 | 0.07 |
|  | |  | |  | **ΣFIC_50_** | **0.79** | **0.08** |
