# Supplementary material for: Screening of the Pandemic Response Box library identified promising compound candidate drug combinations against extensively drug-resistant Acinetobacter baumannii
Source: Sci Rep. 2024 Sep 17;14:21709. doi: 10.1038/s41598-024-72603-9 (PMC11408719; doi:10.1038/s41598-024-72603-9)
Supplement: Supplementary file 4 — Supplementary Table S3. [file 41598_2024_72603_MOESM4_ESM.docx]

**Table S3.** the 50% fractional inhibitory concentrations (FIC_50_) of MUT056399 (Drug A) and eravacyclin (Drug B) combination against *A. baumannii* 5075

| **Drug A : Drug B** | **Sum of FIC's (1st)** | | **Sum of FIC's (2nd)** | | **Sum of FIC's (3rd)** | **Mean** | **SD** |
| --- | --- | --- | --- | --- | --- | --- | --- |
| **RATIOS** | **FIC_50_ Drug A + FIC_50_ Drug B** | | **FIC_50_ Drug A + FIC_50_ Drug B** | | **FIC_50_ Drug A + FIC_50_ Drug B** |  |  |
| 1 to 1 | 1.003 | | 0.960 | | 0.886 | 0.950 | 0.06 |
| 1 to 3 | 1.146 | | 0.556 | | 1.005 | 0.903 | 0.31 |
| 3 to 1 | 0.775 | | 0.893 | | 0.659 | 0.776 | 0.12 |
| 1 to 4 | 0.919 | | 1.012 | | 0.591 | 0.841 | 0.22 |
| 4 to 1 | 0.707 | | 0.955 | | 0.764 | 0.809 | 0.13 |
| 1 to 2 | 1.012 | | 1.084 | | 0.989 | 1.028 | 0.05 |
|  | |  | |  | **ΣFIC_50_** | **0.884** | **0.06** |
