# Supplementary material for: Screening of the Pandemic Response Box library identified promising compound candidate drug combinations against extensively drug-resistant Acinetobacter baumannii
Source: Sci Rep. 2024 Sep 17;14:21709. doi: 10.1038/s41598-024-72603-9 (PMC11408719; doi:10.1038/s41598-024-72603-9)
Supplement: Supplementary file 5 — Supplementary Table S4. [file 41598_2024_72603_MOESM5_ESM.docx]

**Table S4.** Summary of the concentration used in time-kill assay and biofilm formation assay

Combination of MUT056399 and brilacidin

| **Compound** | **MIC (μM)** | **MUT056399:Brilacidin** | | **Final concentration of MUT0563699 (μM)** | **Final concentration of Brilacidin (μM)** |
| --- | --- | --- | --- | --- | --- |
| MUT056399 | >160* |  |  | |  |
| Brilacidin | 10 |  |  | |  |
|  |  | 1:2 | 1/3 x (100) = 33.33 | | 2/3 x (10) = 6.67 |
|  |  | 4:1 | 4/5 x (100) = 80 | | 1/5 x (10) = 2 |

*If the MIC is greater than 100 μM, the concentration of 100 μM is used for the calcuation.

Combination of MUT056399 and Eravacyclin

| **Compound** | **MIC (μM)** | **MUT056399:Eravacycline** | | **Final concentration of MUT0563699 (μM)** | **Final concentration of Eravacycline (μM)** |
| --- | --- | --- | --- | --- | --- |
| MUT056399 | >160* |  |  | |  |
| Eravacycline | 1.25 |  |  | |  |
|  |  | 1:2 | 1/3 x (100) = 33.33 | | 2/3 x (1.25) = 0.83 |
|  |  | 3:1 | 3/4 x (100) = 75 | | 1/4 x (1.25) = 0.31 |

*If the MIC is greater than 100 μM, the concentration of 100 μM is used for the calcuation.
